# Episodes of Febrile Urinary Tract Infections Occur More Often in the Winter in Patients with Spina Bifida

**DOI:** 10.5152/tud.2023.22190

**Published:** 2023-05-01

**Authors:** Takeya Kitta, Yukiko Kanno-Kakibuchi, Hiroki Chiba, Madoka Higuchi, Mifuka Ouchi, Mio Togo, Yui Abe-Takahashi, Mayuko Tsukiyama, Nobuo Shinohara

**Affiliations:** 1 Department of Renal and Urologic Surgery, Asahikawa Medical University, Asahikawa, Japan; 2 Department of Urology, University of Yamanashi, Graduate School of Medical Sciences, Yamanashi, Japan; 3 Department of Renal and Genitourinary Surgery, Hokkaido University, Graduate School of Medicine, Sapporo, Japan

**Keywords:** Spina bifida, febrile urinary tract infections, seasonality, neurogenic bladder, clean intermittent catheterization

## Abstract

**Objective:**

Febrile urinary tract infections, which commonly occur in spina bifida patients, can cause renal dysfunction. To help prevent febrile urinary tract infection occurrence, a better understanding of any seasonal tendencies would be beneficial.

**Materials and Methods:**

Study points evaluated included: (i) with or without febrile urinary tract infections, (ii) type of urinary management in patients with febrile urinary tract infections, (iii) number of febrile urinary tract infection occurrences, and (iv) season associated with episode. Febrile urinary tract infection was defined by medical records specifically ascribing the term and clinical presentations consistent with the diagnosis. We evaluated febrile urinary tract infection incidence per 1 person, risk odds, expected values, and chi-square analysis.

**Results:**

This study examined 140 patients (79 males, 61 females). The patient's age at the first visit ranged from 2 days to 43.7 years old (median: 3.0 years old). The observation period was 0.6-43.7 years (median: 11.5 years). (i) Febrile urinary tract infection occurred in 68 cases, (ii) urinary management included: full clean intermittent catheterization: 49 cases, autoaugmented bladder: 15 cases, self-voiding: 8 cases, clean intermittent catheterization + indwelling catheter at night time: 5 cases, self-voiding + clean intermittent catheterization: 4 cases, vesicocutaneostomy: 2 cases, (iii) number of febrile urinary tract infection episodes: 2 times or less: 40 cases, 3-5 times: 20 cases, over 6 times: 8 cases, and (iv) total number of febrile urinary tract infection episodes was 183, with spring: 41, summer: 44, autumn: 37, and winter: 61. Risk odds of the incidence (one season vs. the other season) were spring: 0.870 (*P* = .425), summer: 0.954 (*P* = .784), autumn: 0.755 (*P* = .120), and winter 1.497 (*P* = .009).

**Conclusion:**

There is a significantly high incidence of febrile urinary tract infection in spina bifida patients in winter versus the other seasons.

**Main Points**

- Spina bifida patients commonly have febrile urinary tract infections (f-UTI) that can lead to subsequent severe impairment of renal function.
- This study investigated the incidence of f-UTI in spina bifida patients and to determine if there was an association between seasonality and f-UTI episodes, and if so, what the magnitude of this seasonality was.
- We have a significantly high incidence of f-UTI in spina bifida patients in winter rather than in other seasons.

## Introduction

Spina bifida is the most common major birth defect seen in newborns, with an occurrence of 1 per 2000 births.^1^ Spina bifida arises from incomplete development of the neural tube. It is commonly used as a nonspecific term depending on any degree of neural tube closure (subdivided into spina bifida occulta and spina bifida aperta). Spinal dysraphism is a more generic term. In this report, we use spina bifida for neural tube defects following past reports.

Due to advancements in the medical management of this disorder, many spina bifida patients now survive into early adulthood and beyond. Even so, the majority of these patients are known to have neurogenic bladder with a potential risk of urinary tract infection, urinary incontinence, and renal failure along with a poor quality of life. In addition, spina bifida patients commonly have febrile urinary tract infections (f-UTI) that can lead to subsequent severe renal dysfunction. The management goal in spina bifida patients with neurogenic bladder is to preserve renal function. However, similar to the characteristics that are seen for many infectious diseases of public health importance, it has been suggested that seasonality, in which there is a periodic surge in the incidence of the disorder that corresponds to different seasons or specific calendar periods, can play a role in these patients. Even so, there is conflicting evidence regarding the seasonality of UTIs in the literature. In our routine practice, we noticed that we treated more spina bifida patients for f-UTI during certain periods of the year and not equally throughout the year. Thus, the present study attempted to determine if there was an association between seasonality and f-UTI episodes, and if so, what the magnitude of this seasonality was.

## Materials and Methods

This study was approved by the Ethics Committee of Hokkaido University. The study protocol followed the guidelines of the Declaration of Helsinki and was approved by the Ethics Committee of our institute (Institutional Review Board no.: 020-0093). This study was conducted in a retrospective manner, and all data were retrospectively investigated based on the patient’s electronic medical charts done with IRB permission. This is not a forward-looking clinical trial, and informed consent from all patients was not required. Hokkaido University Hospital is in an area in the northern part of our country, and all patients come to the hospital from the surrounding area. We evaluated 140 patients (79 males, 61 females) who had been treated for more than 6 months. The patient's age at the first visit ranged from 2 days to 43.7 years old (median: 3.0 years old). The observation period was 0.6-43.7 years (median: 11.5 years). The study points examined included: (i) with or without f-UTI, (ii) type of urinary management in patients with f-UTI at the latest visit, (iii) the number of occurrences of f-UTI, and (iv) the season associated with the episode (spring—March to May; summer—June to August; autumn—September to November; winter—December to February). All of these points were retrospectively investigated based on the patient’s electronic medical charts. Patients were considered to have f-UTI if they were being administered treatment for f-UTI; they exhibited any signs of f-UTI, such as back pain, scrotum pain, and high-grade pyuria with fever; or when there were any notations in their medical records describing the fever as f-UTI. We calculated the incidence of f-UTI based on 1 person (incidence of f-UTI based on each person), the risk odds, expected values, and the results of the chi-square analysis. And we performed univariate analysis as predictors of f-UTI in patients.

## Results

There were 68 patients (48.6%) who had at least 1 episode of f-UTI, with a total of 183 episodes. The number of episodes was 1 or 2 in 40 patients (58.8%), 3 to 5 in 20 patients (29.4%), and more than 5 in 8 patients (11.8%). Urinary management in the f-UTI patients included: self-voiding: 8 cases (11.8%), self-voiding with clean intermittent catheterization (CIC): 4 cases (5.9%), CIC alone: 49 cases (72.1%), CIC + retained catheter at nighttime: 5 cases (7.4%), and vesicocutaneous fistula: 2 cases (0.9%), with history of autoaugmentation in 15 cases (22.1%). And patients who do not have f-UTI are also summarized in Table 1.

**Figure 1.**
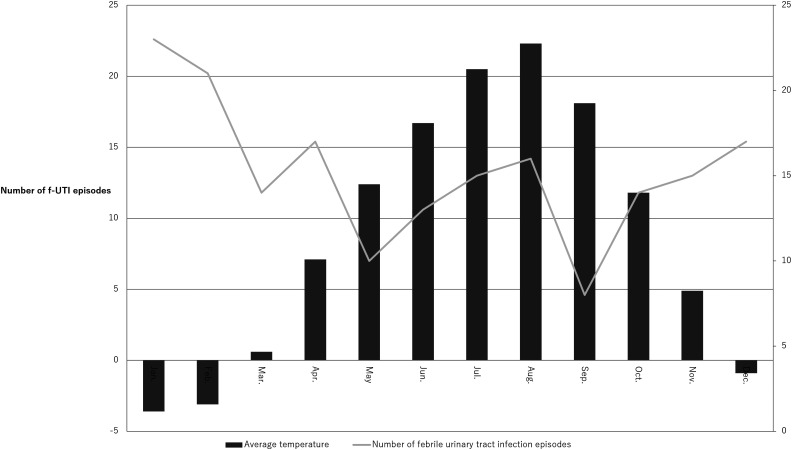
Association between febrile urinary tract infection incidence and temperature. This bar graph and line graph display the relationship between the incidence of febrile urinary tract infections and the average monthly temperature.

Figure 1 shows the relationship between f-UTI occurrences and the average monthly temperature within our geographical region. Febrile urinary tract infection episodes and incidence were higher in the winter (61; 0.0348) versus the spring (41; 0.0235), summer (41; 0.0252), and autumn (37; 0.0210), with the odds ratio of the incidence relative to all other seasons calculated to be 1.497 (*P* = .009), 0.870 (*P* = .425), 0.954 (*P* = .784), and 0.755 (*P* = .120), respectively (Tables 2 and 3). There was a significant positive association between CIC alone (4.053 (2.032, 8.116)), self-voiding (0.2841 (0.1214, 0.7085)), history of augmentation surgery (3.792 (1.351, 9.899)) and f-UTI occurrences (Table 4).

## Discussion

Spina bifida patients exhibited a significantly higher incidence of f-UTI in the winter versus the other seasons. To the best of our knowledge, f-UTI seasonality in spina bifida patients has not been previously reported in the literature.

Filler et al^2^ examined children with spina bifida and reported finding that 50% had their first UTI by 15 months of age, with 44% of the patients having >5 UTI episodes by the time they were 15 years old. Furthermore, the annual incidence of UTI in patients with neurogenic bladder has been reported in other studies to be as high as 20%. Armour et al^3^ examined spina bifida patients for UTI admissions and reported a rate of 22.8 per 1000 patients, whereas patients without spina bifida had a UTI admission rate of 0.44 per 1000 patients. In addition, Caterino et al^4^ reviewed emergency department admissions of spina bifida patients and reported that the most common diagnoses included UTI. In the present study, we found that 68 patients (48.6%) had at least 1 f-UTI episode, which was similar to that reported in the previous studies.

In children with neurogenic bladder, starting from the 1970s, CIC has been widely recommended. Its use has proved to be an important advance in helping to decrease UTI and renal failure in these children. In the present study, CIC was performed in 85.4% of the patients. As spina bifida patients generally require some type of catheterization (CIC and indwelling) for bladder emptying, these patients commonly develop bacteriuria, leukocyturia, hematuria, and positive nitrite. However, normally these children do not usually require any antibiotic therapy, provided there are no additional symptoms present, such as worsening of fever, rigors, or lower urinary tract symptoms, among others.^5^ There is an increased morbidity and mortality found in conjunction with catheter-associated UTIs, with secondary bloodstream infections collectively the most common cause of these UTIs. In the present study, 7.4% of patients used the catheter only at nighttime, with these patients instructed not to use an indwelling catheter for more than 12 hours.

Urinary tract infections are considered to be one of the most common bacterial infections. Foxman et al^6^ reported that by the age of 24, approximately one-third of all women have had at least 1 UTI. It has also been reported that some bacterial infections, such as bacterial meningitis, appear to exhibit a winter seasonal pattern,^7^ while other infections, such as *Campylobacter* and *Salmonella* infections, have been shown to be more common during the warmest months of the year.^8^ Another study has shown that 70%-80% of UTI cases involved *Escherichia coli*.^9^ In addition, *E. coli* bloodstream infections have recently been reported to be associated with a seasonal variation.^10^ On the contrary, Kito et al^11^ reported that there was no seasonal trend in detecting the frequency of isolating *E. coli*. At the present time, there is some conflict in the literature with regard to the seasonality of UTIs, as some studies have shown that the UTI incidence was higher in the winter,^12^ some in the autumn,^13^ and others in the summer.^14^ It is possible that these differences could be due in part to poor methodology, seasonality assessments done in different geographical areas, or perhaps due to differences in age, sex, and community. In the present study, our results showed that the peak of the f-UTI incidence occurred during the winter (December to February). And in this study, we could not confirm all pathogens in all patients. At least, *E. coli* was confirmed the most, and *Klebsiella*, *Pseudomonas*, *Campylobacter*, etc. were also confirmed. Statistical analysis was not performed because of small number. Due to limited Activities of Daily Living (ADL), patients with spina bifida require more time to put on and take off their clothes during the winter. Furthermore, individuals who require assistance, such as self-catheterization, may take longer to begin voiding, compared to the summer season. There was a significant positive association between CIC alone type voiding (4.053 (2.032, 8.116)) and f-UTI occurrences. However, it is difficult to confirm this point. The precise processes behind the seasonality of f-UTI have yet to be established.

We performed univariate analysis as predictors of f-UTI in patients (Table 4). A positive association was observed between CIC alone, self-voiding, history of augmentation surgery and f-UTI occurrences. Regarding the urinary management method, it is thought that more severer the neurological abnormality, the more patients that induce CIC and need to augmentation surgery. In this study, the reason why the self-voiding patients had more f-UTI risk is that the self-voiding patients may have had inappropriate urination management. However, it is difficult to determine the exact cause. Further speculation is difficult because the degree of neurological abnormalities has not been accurately assessed.

Increases in the temperature during the summertime can potentially lead to dehydration in some individuals. It has been suggested that dehydration can increase the risk of UTI, by causing lower urine flow rates and voiding frequencies, which can subsequently delay bacterial ejection from the bladder.^15^ Furthermore, Freeman et al^16^ additionally reported finding increases in temperature led to behavioral changes and more frequent sexual activity, which could be one reason for an increased risk of UTI.^16^ The present study was not able to confirm this speculation. However, the seasonal peak found for f-UTI in the present study differs from the findings reported in these previous reports. Therefore, the influence of dehydration and temperature on UTI incidence remains unclear.

At the present time, there have yet to be widely published, definitive diagnosis and management guidelines for UTI in spina bifida patients. Thus, since insights on the evaluation and treatments of UTI are not well known in this patient population, clinicians will often apply general pediatric guidelines. However, guidelines for the diagnosis, evaluation, and treatment of UTIs in children have been developed by the American Academy of Pediatrics. Although these guidelines do work well when children have normal urinary tract function, in the spina bifida population, the urologic complexity of these patients makes universal application of these practices difficult to enact. When a group of physicians who routinely treat spina bifida patients at specialty centers were surveyed, results demonstrated that there were differences in the data interpretation with regard to diagnosing and managing UTIs.^17^

To our knowledge, there are no f-UTI incidence time series available for spina bifida patients that can be used to test for the seasonality of f-UTI. However, being able to determine the seasonality dynamics of f-UTI and the potential association with the determinants of infection would be of great benefit to these patients. Moreover, this information would also help clinicians and infection control specialists to better understand the infection risk factors, thereby ultimately helping to improve strategies for preventing these infections.

There were a few limitations to the present study. First, this was a retrospective study that had a relatively small sample size. Second, since our department is a highly specialized university neuro-urology center, there was a potential selection bias, as we could have included more severe cases than would be normally seen in the general population with regard to spina bifida cases. Third, in some patients, it is difficult to differentiate between asymptomatic significant bacteriuria and f-UTI. We consider a patient to have f-UTI if there has been a previous treatment for f-UTI, if the patient has ever reported any signs of f-UTI, such as back pain, scrotum pain, and high-grade pyuria with fever, or when there is a previous medical history that has described the fever as f-UTI. It could not be denied that the definition used in this study may lead to different results from those used in other studies.

In conclusion, although f-UTI of spina bifida patients has multifactorial etiologies, there was a significantly higher incidence of f-UTI observed in spina bifida patients in the winter as compared to other seasons. Future studies with a larger sample size will need to be conducted in order to validate the results of the present study.

## Figures and Tables

**Table 1.** Urinary Management in the Patient with f-UTI and Without f-UTI

Urinary Management	Number (Duplicate)	
	Outbreak of f-UTI (68 Patients)	No Infections (72 Patients)
Full CIC	49	28
Augmented bladder	15	5
Self-voiding	8	23
CIC + indwelling catheter at nighttime	5	3
Self-voiding + CIC	4	9
Vesicocutaneostomy	2	0

CIC, clean intermittent catheterization; f-UTI, febrile urinary tract infections.

**Table 2.** Incidence of f-UTI Per 1 Person, Risk Odds, Expected Values, and the Chi-Square Analysis

		Odds Ratio (95% CI)	*P*
Spring vs.	Summer	1.074 (0.751-1.535)	.740
	Autumn	0.895 (0.616-1.299)	.620
	Winter	1.481 (1.062-2.040)	.047^*^
Summer vs.	Autumn	0.833 (0.577-1.203)	.407
	Winter	1.379 (0.996-1.909)	.097
Autumn vs.	Winter	1.655 (1.175-2.332)	.013^*^

f-UTI, febrile urinary tract infections.

We calculated the incidence of f-UTI per 1 person, risk odds, expected values, and chi-square analysis.

^*^P-value < .05.

**Table 3.** Incidence of f-UTI Per 1 Person, Risk Odds, Expected Values, and the Chi-Square Analysis

		Odds Ratio (95% CI)	*P*
Spring VS.	Summer/Autumn/Winter	0.870 (0.650-1.165)	.425
Summer VS.	Autumn/Winter/Spring	0.954 (0.718-1.268)	.784
Autumn VS.	Winter/Spring/Summer	0.755 (0.714-0.798)	.120
Winter VS.	Spring/Summer/Autumn	1.497 (1.438-1.559)	.009^*^

f-UTI, febrile urinary tract infections.

We calculated the incidence of f-UTI per 1 person, risk odds, expected values, and chi-square analysis.

^*^P-value < .05.

**Table 4.** Univariate Analysis as Predictors of f-UTI in Patients

	OR	95% CI	*P*
Sex	1.359	0.6761-2.615	.3700
Full CIC	4.053	2.032-8.116	<.0001^*^
Self-voiding	0.2841	0.1214-0.7085	.0041^*^
CIC + indwelling catheter at night time	1.825	0.4538-7.089	.4169
Self-voiding + CIC	0.4375	0.1438-1.507	.1775
History of augmentation surgery	3.792	1.351-9.899	.0106^*^

CIC, clean intermittent catheterization; f-UTI, febrile urinary tract infections; OR, odds ratio.

We performed univariate analysis as predictors of f-UTI in patients.

^*^P value < .05.

## References

[1] Shaer CM Chescheir N Schulkin J . Myelomeningocele: a review of the epidemiology, genetics, risk factors for conception, prenatal diagnosis, and prognosis for affected individuals. Obstet Gynecol Surv. 2007;62(7):471 479. (10.1097/01.ogx.0000268628.82123.90) 17572919

[2] Filler G Gharib M Casier S Lödige P Ehrich JH Dave S . Prevention of chronic kidney disease in spina bifida. Int Urol Nephrol. 2012;44(3):817 827. (10.1007/s11255-010-9894-5) 21229390

[3] Armour BS Ouyang L Thibadeau J Grosse SD Campbell VA Joseph D . Hospitalization for urinary tract infections and the quality of preventive health care received by people with spina bifida. Disabil Health J. 2009;2(3):145 152. (10.1016/j.dhjo.2009.02.001) 21122753

[4] Caterino JM Scheatzle MD D'Antonio JA . Descriptive analysis of 258 emergency department visits by spina bifida patients. J Emerg Med. 2006;31(1):17 22. (10.1016/j.jemermed.2005.09.005) 16798148

[5] European Association of Urology. EAU guidelines on urological infections/; 2020. Available at: https://uroweborg/guideline/urological-infections.

[6] Foxman B Barlow R D'Arcy H Gillespie B Sobel JD . Urinary tract infection: self-reported incidence and associated costs. Ann Epidemiol. 2000;10(8):509 515. (10.1016/s1047-2797(00)00072-7) 11118930

[7] Paireau J Chen A Broutin H Grenfell B Basta NE . Seasonal dynamics of bacterial meningitis: a time-series analysis. Lancet Glob Health. 2016;4(6):e370 e377. (10.1016/S2214-109X(16)30064-X) 27198841 PMC5516123

[8] Yun J Greiner M Höller C Messelhäusser U Rampp A Klein G . Association between the ambient temperature and the occurrence of human *Salmonella* and *Campylobacter* infections. Sci Rep. 2016;6:28442. (10.1038/srep28442) PMC4914963 27324200

[9] Stamm WE Hooton TM . Management of urinary tract infections in adults. N Engl J Med. 1993;329(18):1328 1334. (10.1056/NEJM199310283291808) 8413414

[10] Richet H . Seasonality in Gram-negative and healthcare-associated infections. Clin Microbiol Infect. 2012;18(10):934 940. (10.1111/j.1469-0691.2012.03954.x) 22784293

[11] Kito Y Kuwabara K Ono K , et al. Seasonal variation in the prevalence of Gram-negative bacilli in sputum and urine specimens from outpatients and inpatients. Fujita Med J. 2022;8(2):46 51. (10.20407/fmj.2021-003) 35520292 PMC9069267

[12] Stansfeld JM . Clinical observations relating to incidence and aetiology of urinary-tract infections in children. Br Med J. 1966;1(5488):631 635. (10.1136/bmj.1.5488.631) 5908707 PMC1843902

[13] Vorland LH Carlson K Aalen O . Antibiotic resistance and small R plasmids among *Escherichia coli* isolates from outpatient urinary tract infections in northern Norway. Antimicrob Agents Chemother. 1985;27(1):107 113. (10.1128/AAC.27.1.107) 3885842 PMC176214

[14] Rossignol L Pelat C Lambert B Flahault A Chartier-Kastler E Hanslik T . A method to assess seasonality of urinary tract infections based on medication sales and Google trends. PLoS One. 2013;8(10):e76020. (10.1371/journal.pone.0076020) PMC3808386 24204587

[15] Beetz R . Mild dehydration: a risk factor of urinary tract infection? Eur J Clin Nutr. 2003;57(suppl 2):S52 S58. (10.1038/sj.ejcn.1601902) 14681714

[16] Freeman JT Anderson DJ Sexton DJ . Seasonal peaks in *Escherichia coli* infections: possible explanations and implications. Clin Microbiol Infect. 2009;15(10):951 953. (10.1111/j.1469-0691.2009.02866.x) 19845705

[17] Elliott SP Villar R Duncan B . Bacteriuria management and urological evaluation of patients with spina bifida and neurogenic bladder: a multicenter survey. J Urol. 2005;173(1):217 220. (10.1097/01.ju.0000146551.87110.f4) 15592079

